# Global, local and focused geographic clustering for case-control data with residential histories

**DOI:** 10.1186/1476-069X-4-4

**Published:** 2005-03-22

**Authors:** Geoffrey M Jacquez, Andy Kaufmann, Jaymie Meliker, Pierre Goovaerts, Gillian AvRuskin, Jerome Nriagu

**Affiliations:** 1 BioMedware, Inc., 516 North State Street, Ann Arbor, MI, 48104-1236, USA; 2 Department of Environmental Health Sciences, The University of Michigan School of Public Health, 109 S. Observatory St. Ann Arbor, MI, 48109-2029, USA

## Abstract

**Background:**

This paper introduces a new approach for evaluating clustering in case-control data that accounts for residential histories. Although many statistics have been proposed for assessing local, focused and global clustering in health outcomes, few, if any, exist for evaluating clusters when individuals are mobile.

**Methods:**

Local, global and focused tests for residential histories are developed based on sets of matrices of nearest neighbor relationships that reflect the changing topology of cases and controls. Exposure traces are defined that account for the latency between exposure and disease manifestation, and that use exposure windows whose duration may vary. Several of the methods so derived are applied to evaluate clustering of residential histories in a case-control study of bladder cancer in south eastern Michigan. These data are still being collected and the analysis is conducted for demonstration purposes only.

**Results:**

Statistically significant clustering of residential histories of cases was found but is likely due to delayed reporting of cases by one of the hospitals participating in the study.

**Conclusion:**

Data with residential histories are preferable when causative exposures and disease latencies occur on a long enough time span that human mobility matters. To analyze such data, methods are needed that take residential histories into account.

## Background

U.S. population-based surveys estimate that adults spend 87% of their day indoors, 69% in their place of residence, and 6% in a vehicle [1-3]. To date, most published disease cluster investigations use static geographies in which individuals are assumed to be sessile. Examples include the use of geocoded place of residence at time of diagnosis, death, and at time of birth (e.g. [4]), as well as the address of the admitting hospital (e.g. [5]) to record locations of health events, even though most researchers acknowledge that residential mobility should be accounted for, especially for diseases with long latencies such as cancer. In a recent review of standard methods for evaluating exposure/hazards, disease mapping and clustering techniques, Bayesian approaches, Markov Chain Monte Carlo (MCMC) and geostatistical methods, Mather et al. [6] identified as substantial weaknesses (1) the lack of temporal referencing of geospatial data and (2) the inability of methods to account for residential histories. A recent meeting of this nation's experts recognized the need to account for latency and human mobility as especially pressing in studies of cancer [7]. Boscoe et al. [8] identified residential history information as a primary need for the analysis of cancer data.

The representation of individuals as sessile (immobile) rather than vagile (mobile) in part is due to the static world view of GIS software, which is not well suited to representing temporal change [9,10]. Recently, technological advances have resulted in Space Time Intelligence Systems (e.g. [11-13]) that implement several constructs from Geographic Information Science for representing human mobility (see [14] for a review). The methods presented in this paper build on this body of prior work to produce case-control cluster statistics for residential histories.

We begin with a brief background on tests for disease clustering, followed by a summary of approaches to modeling human mobility. We then develop a suite of novel tests for evaluating local, global and focused clustering in residential histories using case-control data. Finally, we illustrate several of the new techniques by quantifying local, global and focused clustering of residential histories in a case-control study of bladder cancer in Michigan.

### Background on cluster tests

Cluster tests work within a hypothesis testing framework that proceeds by calculating a statistic (e.g. clustering metric) to quantify a relevant aspect of spatial pattern in a health outcome (e.g. case/control location, disease incidence, or mortality rate). The numerical value of this statistic is then compared to the distribution of that statistic's value under a null spatial model, providing a probabilistic assessment of how unlikely an observed cluster statistic is under the null hypothesis [15]. Waller and Jacquez [16] formalized this approach by identifying five components of a spatial cluster test. The test statistic quantifies a relevant aspect of spatial pattern (e.g. Moran's *I*). The alternative hypothesis describes the spatial pattern that the test is designed to detect. This may be a specific alternative, such as a circular cluster for the scan statistic, or it may be the omnibus "not the null hypothesis". The null hypothesis describes the spatial pattern expected when the alternative hypothesis is false (e.g. uniform cancer risk). The null spatial model is a mechanism for generating the reference distribution. This may be based on distribution theory, or it may use randomization (e.g. Monte Carlo) techniques. Most disease cluster tests employ heterogeneous Poisson and Bernoulli models for specifying null hypotheses [17]. The reference distribution is the distribution of the test statistic when the null hypothesis is true. Comparison of the test statistic to the reference distribution allows calculation of the probability of observing that value of the test statistic under the null hypothesis of no clustering. This five-component mechanism underpins most commonly used clustering methods.

There are dozens of cluster statistics (see [17-19] for reviews) that may be categorized for convenience as global, local, and focused tests. Global cluster statistics are sensitive to spatial clustering, or departures from the null hypothesis, that occur anywhere in the study area. Many early tests for spatial pattern, such as Moran's *I *[20] are global tests. While global statistics can determine whether spatial structure (e.g. clustering, autocorrelation, uniformity) exists, they do not identify where the clusters are, nor do they quantify how spatial dependency varies from one place to another.

Local statistics such as Local Indicators of Spatial Autocorrelation (LISA) [21] quantify spatial autocorrelation and clustering within the small areas that together comprise the study geography. Local statistics quantify spatial dependency (e.g. not significantly different from the null expectation, cluster of high values, cluster of low values, and high or low spatial outlier) in a given locality. Many local statistics have global counterparts that often are calculated as functions of local statistics. For example, Moran's *I *is the sum of the scaled local Moran statistics.

Focused statistics quantify clustering around a specific location or focus. These tests are particularly useful for exploring possible clusters of disease near potential sources of environmental pollutants. For example, Lawson and Waller [22,23] proposed tests that score each area for the difference between observed and expected disease counts, weighted by exposure to the focus (also see [24] for a review of these approaches). A commonly used exposure function is inverse distance to the focus (1/d). The null hypothesis is no clustering relative to the focus, with expected number of cases calculated as the Poisson expectation using the population at risk in each area and the assumption that risk is uniform over the study area.

Hundreds of cluster investigations are recorded in the literature, and several of these have resulted in cancer control activities such as epidemiological studies to understand potential causes. Notable examples of cluster studies include brain cancer [25], liver cancer [26], breast cancer [27,28], prostate cancer [29], colorectal cancer [30], and cancer disparities [31], to name only a few.

In studies of lung, breast and colorectal cancer on Cape Cod, stronger evidence for spatial clustering was found once latency was taken into account [32]. In a population-based case-control study Vieira et al. [33] incorporated residential location to evaluate lung cancer risks not explained by age and smoking. Han et al. [34] explored geographic clustering of breast cancer based on place of residence early in life and found space-time clustering in case-control data. They also explored clustering of cases using place of residence at critical time points including at the subject's birth, menarche, and at the women's first birth. Boscoe et al. [8] recognized representation of residential mobility as a primary need for data used in studies of cancer. But to date and to our knowledge, residential mobility has yet to be directly accounted for in cluster studies.

How might one account for residential mobility in cluster studies? Hagerstrand [35] conceptualized the *space time path *as an individual's continuous physical movement through space and time, and visually represented this as a 3-dimensional graph. Hornsby and Egenhofer [36] recognized that space-time paths mediate individual-level exposure to pathogens and environmental toxins, and that practical application would require a mechanism for representing location uncertainty. A *space time prism *refers to the possible locations an individual could feasibly pass through in a specific time interval, given knowledge of their actual locations in the times bracketing that interval. The *potential path area *[37] shows the locations the individual could occupy given these constraints, and represents places where exposure events might occur. These constructs enabled new research approaches in diverse fields such as student life [38], sports analysis [39], social systems [40], transportation [37], and the analysis of disparities in gender accessibility in households [41]. While these approaches provide a proven mechanism for modeling geospatial lifelines and related constructs, to date and to our knowledge there are no methods for the statistical evaluation of clustering among such lifelines other than the paper by Sinha and Mark [42], who use Minkowski-type metrics to calculate a dissimilarity metric for geospatial lifelines, and then cluster this dissimilarity metric.

This paper proposes a novel technique for undertaking the statistical evaluation of clustering of residential histories for case-control data. We first develop the method, and then apply it to an ongoing case-control study of bladder cancer in southeastern Michigan.

### Setting the Problem

A naïve approach when considering residential histories is to take an existing test for spatial clustering, and to then apply it repeatedly for different time values. For example, when considering the geographic distribution of bladder cancer, one might use place of residence of individuals in a case-control study from *T *years ago, and then allow *T *to vary in a range of several decades. Locations of place of residence will change, as may the numbers of cases and controls extant in the study area. How might results vary depending on when one looks at the system (e.g. on selection of *T*)? To answer this question we analyzed data from a population-based bladder cancer case-control study currently underway in southeastern Michigan. Cases are recruited from the Michigan State Cancer Registry and diagnosed in the years 2000–2004.

**Figure 1:**
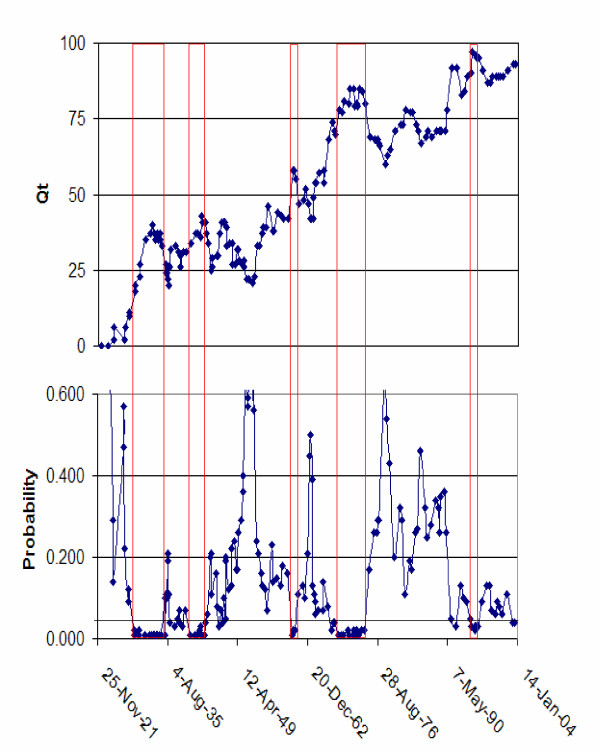
**Cuzick and Edward's statistic through time**. Graph of Cuzick and Edward's T_k _statistic (top) and its Probability (bottom) through time for *k *= 5. Shown in red are those time intervals in which the probability of T_k _was 0.0l or smaller.

Controls are frequency matched to cases by age (± 5 years), race, and gender, and recruited using a random digit dialing procedure from an age-weighted list. This data set is described more fully later in this paper, and is comprised of 63 cases and 182 controls. Using Cuzick and Edwards *T*_*k *_statistic with *k *= 5 nearest neighbors we then analyzed these data at every point in time when the topology of place of residence of the cases and controls changed by having a participant move, enter or leave the study area. The graph of *T*_*k *_through time (Figure 1) is ascending, reflecting the larger number of cases and controls residing in the study area in later time periods. We found five periods when cases were significantly clustered relative to the controls: January 1 1929 through January 1 1935, January 1 1941 through November 26 1942, January 1 1960 through January 1 1961, August 22 1967 through January 1 1975 and January 1 1995 through January 1 1997. Clearly, results of cluster analyses that rely on single locations may be highly sensitive to the choice of the time at which the analysis is conducted. What are needed are new methods that account for the dynamic topology of cases and controls that arise as a consequence of residential mobility, and that are suited to multi-temporal analyses. The development of such techniques is the focus of this paper.

## Methods

We begin by defining an algebra for residential histories, and a matrix representation that describes how spatial nearest neighbor relationships change through time. Next we develop a local case control cluster test, and then extend it to create global, local and focused tests at specific time points, and then for entire residential histories. After completing development of the cluster statistics for residential histories, we next describe exposure traces that account for latency periods and exposure windows. We then develop clustering methods for exposure traces. After that we describe the bladder cancer data set that was analyzed with the new methods. In the Results section we describe application of several of the new cluster tests to evaluate possible clustering of residential histories of cases of bladder cancer in Michigan.

### Notation

Define the coordinate **u**_*i*,*t *_= {*x*_*i*,*t*_, *y*_*i*,*t*_} to indicate the geographic location of the place of residence of the *i*^th ^case or control at time *t*. Residential histories for individual cases and controls can then be represented as the set of space-time locations:

**L**_*i *_= (**u**_*i*0_, **u**_*i*1_,..., **u**_*iT*_)     (Equation 1)

**Figure 2:**
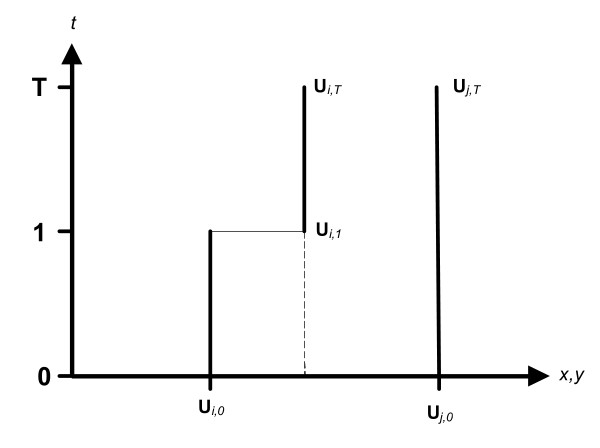
**Schematic of residential histories**. Graphical representation of residential histories from Equation 1 using the instantaneous displacement movement model. Location is on the *x*-axis, time on the *y*-axis. Individual *i *moves from location **u**_*i*0 _to **u**_*i*1 _at time *t *= 1, and stays at that place of residence until *t *= T. Individual *j *stays at the same place of residence from *t *= 0 to *t *= T.

This defines individual *i *living at his or her place of residence found at **u**_*i*0 _at the beginning of the study (time 0), and moving to location **u**_*i*1 _at time *t *= 1. At the end of the study individual *i *may be found at **u**_*iT*_. *T *is defined to be the number of unique observation times on all individuals in the study. This bears some emphasis as understanding of how *T *is recorded is essential in order to understand the cluster tests for residential histories. In other words, *T *is the total number of different observation times across all individuals, and so one might expect several geographic locations in an individual residential history to be the same. For example, suppose we have 2 individuals (*i *and *j*) and record their residential histories (Figure 2). We record their places of residence at *t *= 0, the beginning of the study. At some time *t *= 1 "*i*" moves to a different home, and moves again at time *t *= 2. "*j*" never moves at all and hence has the location of the same initial place of residence recorded at times *t *= 0, 1, and 2. In this example *T *= 2. Notice the duration between *t *= 0 to *t *= 1 may not equal the duration from *t *= 1 to *t *= 2. This will be important later when we develop duration-weighted versions of the statistics.

While observations on residential histories occur at a finite number of time points or observation times, these observations do not have to happen at the same time for all individuals under scrutiny. When residential histories are self-reported, these observation times are defined by the "move" dates reported by the respondent. We modeled this as an instantaneous displacement from the spatial coordinates for entity *i *at time *t *(**u**_*it*_) to those at time *t*+1 (**u**_*it*+1_). We defined this instantaneous displacement as occurring at time *t*+1. We viewed this as an observational model in which the entity is assumed to reside at its known location up until that moment when it is observed elsewhere (e.g. Figure 2).

Individual residential histories can be associated with time-dependent attributes such as weight, height, disease state, smoking status, case control status, and so on. These attributes may be associated with risk and thereby influence calculation of the latency period and exposure windows defined later. Later we also will use time of diagnosis to define exposure windows during which carcinogenesis was thought to have occurred. For now let us define a case-control identifier, *c*_*i *_to be



Define n_a _to be the number of cases and n_b _to be the number of controls. The total number of individuals in the study is then *N *= *n*_*a*_+*n*_*b*_.

### Nearest Neighbor Relationships

Let *k *indicate the number of nearest neighbors to consider when evaluating nearest neighbor relationships (e.g. [63]), and define a nearest neighbor indicator to be:



We then define a binary matrix of *k*^th ^nearest neighbor relationships at a given time *t *as:



By convention we define *η*_*i*,*i*,*k*,*t *_= 0 (the diagonal elements) since we do not wish to count individuals as nearest neighbors of themselves. This matrix enumerates the *k *nearest neighbors (indicated by a 1) for each of the *N *individuals. The entries of this matrix are 1 (indicating that *j *is a *k *nearest neighbor of *i *at time *t*) or 0 (indicating *j *is not a *k *nearest neighbor of *i *at time *t*). It may be asymmetric about the 0 diagonal since nearest neighbor relationships are not necessarily reflexive (e.g. Imagine 3 people, call them A, B and C, standing in a line. B is in the middle but is closer to person A than to person C. The nearest neighbor to C is B, but the nearest neighbor to B is A. The nearest neighbor relationships are not reflexive). Since two individuals cannot occupy the same location, we assume at any time *t *that any individual has *k *unique *k*-nearest neighbors. (While it is true that two individuals cannot occupy the exact same location, such as the space occupied by one individuals body, residential history information can assign two individuals the same coordinate when they live in the same house. How might tied nearest neighbor relationships arising from this situation be resolved? Two approaches have been proposed. The first creates fractional nearest neighbor weights [43], the second propagates uncertainty in the nearest neighbor relationships by evaluating the permutations of possible nearest neighbors for the tied nearest neighbor relationships [44]). The row sums thus are equal to *k *(*η*_*i*,•,*k*,*t *_= *k*) although the column sums vary depending on the spatial distribution of case control locations at time *t*. The sum of all the elements in the matrix is *Nk*. There exists a 1 × *T*+1 vector of times denoting those instants in time when either (1) the system is observed and the locations of the entities are recorded, or (2) under continuous observation at least one entity changes geographic location. We can then consider the sequence of *T *nearest neighbor matrices given by



This defines the sequence of *k *nearest neighbor matrices for each unique temporal observation recorded in the data set, and thus quantifies how nearest neighbor relationships change through time. This demonstrates one way in which spatial weights (here the nearest neighbor relationship) can be specified from residential histories. We will now use these nearest neighbor relationships to construct case control spatial and space-time cluster tests for residential histories.

### Spatially and Temporally Local Spatial Cluster Statistic

A spatially and temporally local case-control cluster statistic is:



This is the count, at time *t*, of the number of *k *nearest neighbors of case *i *that are cases, and not controls (assuming *i *indeed is a case, if it isn't *Q*_*i*,*k*,*t *_= 0). Since a given individual *i *may have *k *unique nearest neighbors, this statistic is in the range 0..*k*. It always is 0 when *i *is a control. When *i *is a case, low values indicate cluster avoidance (e.g. a case surrounded by controls), and large values (near *k*) indicate a cluster of cases. When *Q*_*i*,*k*,*t *_= *k*, at time *t *all of the *k *nearest neighbors of case *i *are cases.

### Probabilities, Null Hypotheses and Randomization

The statistical significance of *Q*_*i*,*k*,*t *_may be evaluated using conditional randomization that holds the case control identifier for individual *i *fixed and then allocates the vector of remaining *N*-1 case-control identifiers across the remaining individuals with a given probability function. If we assume equiprobability such that all individuals have equal disease risk we obtain:



Given the case-control identifier for individual *i*, this is the probability of individual *j *being a case under Goovaert and Jacquez's [45] neutral model Type IV (H_IV_) of spatial independence of risk for a spatially heterogeneous population density. As expressed in Equation 7, the exact number of cases (*n*_*a*_) and controls (*n*_*b*_) might not be reproduced under probabilistic sampling.

Their neutral model type V retains a specified level of spatial autocorrelation and may be simulated using rejection sampling, sequential indicator simulation, or conditional case-control index swapping to achieve the observed level of spatial autocorrelation [46]. Probabilities for neutral model type V are difficult to write in a closed form analogous to Equation 7.

Probabilities for neutral model type H_VI _describe the situation where not all individuals have the same probability of being labeled a case. This occurs, for example, when we are concerned with detecting clusters that arise from additional risk *above and beyond *that of a background risk that is itself spatially heterogeneous. This may be accomplished in a variety of fashions to model known individual and environmental risk factors. Tests of the significance of *Q*_*i*,*k*,*t *_are then identifying clusters of cases above and beyond that expected under the neutral model.

One calculates the value of the test statistic for each realization of the spatial distribution of cases generated under the chosen neutral model. These values under randomization are retained and used to construct the reference distribution of the statistic under the corresponding null hypothesis. The observed value of the test statistic for the not randomized data (denoted ) is then compared to the reference distribution to calculate the p-value:



Here *a *is the number of conditional randomizations whose cluster statistic was greater than or equal to that observed for the not randomized data, and *b *is the total number of randomization runs conducted.

A convenient algorithm for conditional randomization under neutral model IV is to hold the case-control identifier for the *i*^th ^individual constant, and to then draw from the 1 × N-1 vector of remaining case-control identifiers new case-control identifiers for the *k *nearest neighbors surrounding *i*. This sampling is accomplished without replacement. Alternatively, one could populate the *k*-nearest neighbors about *i *using the probabilities from Equation 7. This equation is correct for the first identifier so drawn, but needs to be adjusted for the second, third and so on. For the *m*^th ^identifier the correct probability for sampling without replacement is:



If one assumes sampling with replacement, so that the cases and controls are assumed drawn from a larger population, one can use Equation 7 without modification.

This approach does not work for neutral models type V and VI, since spatial structure in the background risk is lost. Instead one calculates the value of the test statistic for each of the N locations, for each realization of the spatial neutral model (of type V or VI) that produces a spatial point pattern of cases and controls with the desired level of spatial autocorrelation. The probability assigned to clusters from these tests (as given by Equation 8) then accounts for the specified background variation in disease risk.

Note for each of the approaches listed above, that a reference distribution, test statistic, and corresponding p-value, may be calculated for each of the *n*_*a *_case locations.

### Simes Correction for Local Dependency

The P-values for the *k *individuals surrounding the *i*^th ^case are not independent of one another, as they include one another as their own *k *nearest neighbors. We therefore employ a modified Simes correction [47] to account for the lack of spatial independence in the local Q statistics. The Simes adjustment is calculated as p_i_' = (*k *+ 1 - *a*) p_i_. Here *k *is the number of p-values being considered (the number of neighbors), and *a *is the index (starting at 1) indicating the rank in the sorted vector of p values for individual *i *and its neighbors. We employ this correction later when reporting p-values for the local Q-statistics.

### Global Test for Spatial Clustering at Time *t*

A global statistic for spatial clustering at time *t *may be constructed as:



This is the time-referenced form of Cuzick and Edward's [43] global test for case-control clustering used in Figure 1. It is the count, over all cases, of the number of cases that are *k*-nearest neighbors to those cases at time *t*. One could divide this statistic, and others to follow, by *n*_*a *_to facilitate their interpretation. The test statistic would then be an average number of neighbor cases per case instead of the integer total number of cases, and would facilitate comparison across different studies with different numbers of cases. In this paper we will use the case-count version.

The probability of *Q*_*k*,*t *_under H_IV _is evaluated by allocating the case-control id's with equal probability over the *N *locations at time *t*. *Q*_*k*,*t *_is then calculated and this process is repeated *b *times to construct the reference distribution and probability (Equation 8). Notice that since this is a global test conditional randomization that holds the case-control id for individual *i *constant is not needed.

### Global Test for Spatial Clustering of Residential Histories

A global test for spatial clustering among the *N *residential histories as represented in Equation 1 is



This is the sum, over all *T*+1 time points, of the global statistic *Q*_*k*,*t*_. It is a measure of the persistence of global clustering and is large when case clustering persists through time. Its reference distribution may be constructed under a randomization procedure in which the case-control ids are allocated with equal probability over the residential histories comprising the set

{**L**_*i*_, *i *= 1..*N*}     (Equation 12)

This randomization procedure is conditioned on the total number of cases and controls in the data set, so that each data set constructed under randomization has the same number of cases and controls as the original data.

### Local Test for Spatial Clustering of Residential Histories through Time

To determine whether cases tend to cluster through time around a specific case we may construct a test statistic:



For the *i*^th ^residential history, this is the sum, over all *T*+1 time points, of the local spatial cluster statistic *Q*_*i*,*k*,*t*_. It is the number of cases that are *k*-nearest neighbors of the *i*^th ^residential history (a case), summed over all *T*+1 time points. It will be large when cases tend to cluster around the *i*^th ^case through time. Under neutral model type IV, the significance of *Q*_*i*,*k*,*t *_is evaluated under a conditional randomization that holds the case id for *i *constant, and then allocates the remaining case-control id's at random over the *N*-1 remaining residential histories. This statistic is useful for determining whether there is local clustering of residential histories about a specific case. The statistic can be calculated for all cases in the data set to identify those cases whose residential histories form local spatial clusters. However, when calculating significance one should correct for the multiple testing inherent when many spatial locations are evaluated.

### Focused Test for Spatial Clustering at Time *t*

Suppose that one suspects that the cases may be clustering about a specific focus defined by the lifeline (e.g. record of business addresses):

**L**_*F *_= {**u**_*F*,0_, **u**_*F*,1_,.., **u**_*F*,*T*_}      (Equation 14)

This records the locations of the focus as it moves about through space-time, and includes situations in which the focus doesn't move as a degenerate instance. A test for spatial clustering of cases about the focus at a given time *t *is then:



Here *η*_*F*,*j*,*k*,*t *_is the nearest neighbor index indicating at time *t *whether the *j*^th ^individual is a *k*^th ^nearest neighbor of the geographic location of the focus defined by **u**_*F*,*t*_. The statistic *Q*_*F*,*k*,*t *_is then the count of the number of *k*-nearest neighbors about the focus at time *t *that are cases. Under null hypothesis type IV randomization at time *t *may be accomplished by allocating the case control identifiers with equal probability over the *N*-individuals. Since only the *k*-nearest neighbors are considered it is only necessary to allocate their indices. This may be accomplished by sampling without replacement from the 1 × *N *vector of the case-control identifiers, or by drawing the *k *required case control identifiers with probabilities defined by Equation 9 (for sampling without replacement) or Equation 7 (for sampling with replacement).

### Focused Test for Spatial Clustering of Residential Histories about a Mobile Focus

A test for focused clustering of residential histories through time is:



This is the count, over the *T *times, of the number of cases that are *k *nearest neighbors of the focus at each time point. This statistic is large when residential histories that are near the focus are cases. Its maximal value is

max(*Q*_*F*,*k*_) = *kT*.     (Equation 17)

One drawback of using nearest neighbor relationships for focused tests is that the set of nearest neighbors to the focus are given equal weight in Equations 15 and 16, regardless of their actual geographic distance and direction with respect to the focus. But diffusion and active transport mechanisms that might carry emissions from the focus typically result in higher exposures near the focus, and it thus may make sense to use a maximum distance within which *k*_*i *_nearest neighbors are found. In these instances the set of nearest neighbors to the focus will vary (hence the *i *subscript denoting the *i*^th ^focus) depending on the number of cases and controls found within the specified distance of the focus.

### Power of the Focused Tests and Specification of the Exposure

Notice that the power of the tests given by Equations 15 and 16 decreases as *k *approaches *N *since *Q*_*F*,*k*,*t *_= *n*_*a *_when *k *= *N*, and its probability is then:

*P*(*Q*_*F*,*k*,*t *_| *H*_0_, *k *= *N*) = 1.0.     (Equation 18)

When one wishes to search for clustering in instances where *k *approaches *N *power may be retained by constructing a weight function to model the hypothesized exposure. For geographically localized foci this may be based on proximity to the focus. One choice is



Here *r*_*F*,*j*,*t *_is the rank indicating proximity of the location of the *j*^th ^individual at time *t *(as given by **u**_*j*,*t*_) to the location of the focus at time *t *(**u**_*F*,*t*_). For example, the first nearest neighbor to the focus has rank 1, the second rank 2, and so on.

In many situations, such as airborne pollution or groundwater contamination, the magnitude of exposure is a function not only of the proximity to the focus but also of its orientation, since most dispersion processes (i.e. winds, infiltration through porous media) are anisotropic or direction-dependent. Depending on the amount of information available, exposure models of increasing complexity can be built.

An easy way to account for anisotropy is to replace the rank value *r*_*F*,*j*,*t *_by a function of the separation vector **h**_*jF*,*t *_= |**u**_*j*,*t *_- **u**_*F*,*t*_| joining the location of the *j*th individual at time *t *to the location of the focus at time *t*. Covariance functions seem to be natural choices for the weight functions *w*_*F*,*j*,*t *_since they incorporate the spatial pattern of dependence of exposure data. For example, one could use the Exponential or Gaussian covariance functions defined as:





where a(*θ*) is the practical range of autocorrelation of the covariance models; that is the distance *h *at which the covariance function equals 0.05. This range is a function of the azimuth of the separation vector **h**_*jF*,*t*_. For example, the range of exposure to an airborne contaminant is expected to be larger in the direction of the prevailing winds.

More complex weight functions could be created if a process-based model of dispersion is available. For the example of airborne pollution, an atmospheric dispersion and deposition model could be developed to predict the fate of emissions and dust plumes from targeted facilities [48]. However, such models require many more parameters and assumptions concerning, for example, the emission rate, the meteorological conditions, complex terrain effects, the particle size and density for deposition calculation.

A limitation of process-based models is that they fail to provide a measure of uncertainty attached to their predictions and field exposure data are not readily incorporated. Geostatistics [49] provide tools for modeling the spatio-temporal distributions of exposure and assessing the attached uncertainty. Various sources of information can be taken into account, such as measurements at a few monitoring stations, coordinates of major sources of exposure (i.e. factories) and transport characteristics (i.e. wind directions) that could be either directly incorporated into the prediction algorithm [50] or fed into physical models to derive spatial trends [51]. In the latter case, geostatistics are used to model the unexplained or residual part of the variability predicted by the process-based models.

The weight function, either based on geographic proximity (as in Equation 19) or derived using a process-based model or geostatistics (as in Equations 20 & 21), is then used to construct the weighted focused test at time *t *as:



The test for spatial clustering of residential histories about the focus through time is then:



Notice these weighted tests are conducted for the k nearest neighbors being considered. When *k *= *N *the maximum values are:



### Duration-Weighted Tests for Clustering of Residential Histories

The number of time points defined by the *t *= 0 ..*T *observation times, and the frequency with which they are taken, can have some influence on the value of the above statistics. For example, many repeated observations when there is a chance of clustering could lead to spurious significance for the local and global tests for clustering of residential histories. We therefore developed duration-weighted versions of the tests, and these are presented in the Appendix [see Additional file 1].

### Accounting for Exposure Windows and Latency Periods

When dealing with cancers, causative exposures may occur during an exposure window (Δ_*E*_), followed by a latency period (Δ_*L*_) before cancer is manifested and diagnosed. Given the residential history for case *i*, **L**_*i*_, further denote the space-time coordinate representing place of residence at time of diagnosis as , noting that  ∈ **L**_*i *_We can then define that subset of the residential history **L**_*i *_over which the exposure window occurred as:



Here *t*_*i*,*D *_is the time of diagnosis for individual *i*. The term (*t*_*i*,*D *_- Δ_*L*_) indicates the time prior to diagnosis when the latency period began and (*t*_*i*,*D *_- Δ_*L *_- Δ_*E*_) is the time when the causative exposure began. Hence equation 25 denotes that portion of individual *i*'s residential history where causative exposures could have occurred. Notice that both the exposure window and latency period could be covariate-adjusted to account for risk factors such as smoking and age (see Discussion). In this instance the latency period and exposure window vary from one individual to another and we write:



Here Δ_*i*,*L *_and Δ_*i*,*E *_are the latency period and exposure windows for the *i*^th ^individual. In either case (Equations 25 or 26) we call  the *exposure trace *for the *i*^th ^individual.

### Randomization Procedures for Exposure Traces

In order to evaluate whether exposure traces of the cases cluster we must first construct a randomization procedure for generating representative times of diagnosis, latency periods, and exposure windows. Once this is accomplished we will be able to determine whether the exposure traces for the cases cluster relative to those so constructed for the controls. For a case, the exposure trace is defined by the time of diagnosis and the latency period, with the latency period potentially dependent on age, gender and other covariates. The procedure proceeds as follows:

(1) Since controls are matched to cases, the "time of diagnosis" for each control is set equal to the time of diagnosis for the matched case.

(2) The exposure window and latency period for each control is then defined based on the covariates for each control as was accomplished for that controls matched case.

(3) Completion of steps (1) and (2) will result in exposure traces defined for both cases and controls. Now randomly assign case control identifiers across the residential histories with equiprobability conditioned on the total number of cases and the total number of controls.

(4) Calculate the desired test statistic for clustering of exposure traces.

(5) Repeat steps 3 and 4 a desired number of times to construct the reference distribution of the statistic under randomization.

Test statistics for assessing clustering of exposure traces are presented below.

### Local Case-Control Test for the Spatial Clustering of Exposure Traces at Time *t*

When health events such as cancers are caused by exposure to geographically localized factors we might expect the exposure traces for the cases to cluster relative to the exposure traces that are generated for the controls. The durations of the exposure traces may vary, and we therefore will employ duration-weighted statistics. We would like to know whether exposure traces for the cases exhibit spatial clustering relative to the controls both locally (to identify places where causative exposures occurred) and globally (to ascertain whether the exposure traces for the cases cluster when considered as a group). We also might wish to ask whether exposure traces for the cases exhibit focused clustering.

The exposure trace for case *i *() records those places where that individual lived during that time when exposures occurred that might have caused cancer later in life. Now define an indicator, *e*_*i*,*t*_, as:



When *e*_*i*,*t *_is 1, let us say the exposure trace is "active". A local case-control test for spatial clustering of exposure traces at time *t *is then:



This is the count, at time *t*, of the number of *k *nearest neighbors of case *i*'s active exposure trace that are cases (and not controls) whose exposure traces also are active. Hence the statistic will be large at those times when exposure traces of a group of cases are active and cluster. Its value is 0 when individual *i *is a control, and also when individual *i *is a case with an inactive exposure trace. The duration weighted version of this statistic is:



### Local Case-Control Test for the Spatial Clustering of Exposure Traces through Time

We can explore whether active exposure traces of cases tend to cluster spatially through time. A statistic sensitive to this pattern is:



 will tend to be large when active exposure traces for cases tend to cluster around the active exposure trace of the *i*^th ^case. It will be 0 when *i *is a control, and small when a given case *i *has the traces of many controls as its neighbors. The duration-based version of this statistic is:



This statistic will be expressed in case-time units, indicating the number (for example) of case-days over the entire study period for which cases with active traces were *k*-nearest neighbors of the active trace of case *i*.

### Global Case-Control Test for the Spatial Clustering of Exposure Traces at Time *t*

We can ask whether, as a group, active case traces are spatially clustered relative to the active traces of the controls at a given time *t*. This is accomplished using the statistic:



This is simply the sum, over all cases, of the local statistic for clustering of case exposure traces at time *t*. This statistic will be large when active traces of cases tend to be near one another and small when the active traces of cases tend to have controls as their *k *nearest neighbors. The duration-based version is:



### Global Case-Control Test for the Spatial Clustering of Exposure Traces through Time

A global test for the spatial clustering of the active exposure traces of cases through time is:



This is the sum, over all time periods, of the global cluster test for the clustering of exposure traces. It will be large when global clustering of active exposure traces tends to persist through time. The duration-based version of this statistic is:



### Focused Case-Control Test for the Spatial Clustering of Exposure Traces at Time *t*

We can also ask whether the exposure traces of cases cluster near putative emission sources. Again, these sources may be mobile, and we accomplish this by assigning larger weights for those cases that are near the focus. Recall from Equation 14 that we can represent a mobile source as **L**_*F *_= {**u**_*F*,0_, **u**_*F*,1_,.., **u**_*F*,*T*_}. The test for spatial clustering of cases about a focus at a given time *t *(Equation 15) may then be extended to be a focused test for clustering of exposure traces as:



This is the count of the number of cases with active exposure traces that are *k *nearest neighbors of the focus at time *t*. Significance of this statistic may be evaluated by constructing exposure traces for the controls as described earlier, and by then repeatedly allocating case-control identifiers across the *N *lifelines that are *k *nearest neighbors of the focus in order to construct the reference distribution for . The duration weighted version of this statistic is



### Focused Test for Spatial Clustering of Exposure Traces about a Mobile Focus through Time

We can evaluate whether there is statistically significant clustering of exposure traces of cases about a mobile focus through time using the statistic:



This is the count, over *T*+1 times, of the number of cases that have active exposure traces that are *k *nearest neighbors of the focus at each time point. The maximum value of this statistic is *kT*, and its significance may be evaluated under randomization by reallocating case-control identities over the exposure traces of the cases and controls as described in the previous section. The duration-weighted version of this statistic is:



### Weighted Focused tests for Exposure Traces

The power of the *k*-nearest neighbor based focused test for exposure traces decreases as *k *approaches *N*. Weights such as that suggested in Equations 19–21 may be used to construct a weighted focused test for exposure traces at a given time *t*:



The test for focused clustering of exposure traces through time is then:



The significance of these statistics is evaluated using randomization across the *k *nearest neighbors of the focus as described earlier. The corresponding duration-weighted versions are



This is the weighted focused test over duration *ω*_*t*_. The duration-based weighted focused test for exposure traces through time is



### Bladder Cancer in southeastern Michigan

A population-based bladder cancer case-control study is currently underway in southeastern Michigan. Cases are recruited from the Michigan State Cancer Registry and diagnosed in the years 2000–2004. Controls are frequency matched to cases by age (± 5 years), race, and gender, and recruited using a random digit dialing procedure from an age-weighted list. To be eligible for inclusion in the study, participants must have lived in the eleven county study area for at least the past 5 years and had no prior history of cancer (with the exception of non-melanoma skin cancer). Participants are offered a modest financial incentive and research is approved by the University of Michigan IRB-Health Committee.

The data presented here are from 63 cases and 182 controls. As part of the study, participants complete a written questionnaire describing their residential mobility history. The duration of residence and exact street address were obtained, otherwise the closest cross streets were provided. Each residence in the study area was geocoded and assigned a geographic coordinate in ArcGIS; residences outside the study area were not geocoded. Participants resided at 1004 homes within the study area, with time spent averaging 64% of their lifetimes. Residences within the study area were successfully geocoded: 76% automatically matched using ArcGIS settings of spelling sensitivity equal to 75, minimum candidate score equal to 10, and a minimum match score equal to 60. The unmatched addresses were manually matched using cross streets with the assistance of internet mapping services (15%). If cross streets were not provided, best informed guess placed the address on the road (5%), and as a last resort, residence was matched to town centroid (4%).

Industrial histories have also been collected for the study area, and will be explored to explain local clustering. Industries reported to or believed to emit contaminants that have been associated with bladder cancer were identified using the Toxics Release Inventory [52] and the Directory of Michigan Manufacturers (Manufacturer Publishing Co., 1946, 1953, 1960, 1969, 1977, 1982). Standard Industrial Classification (SIC) codes were adopted, but prior to SIC coding, industrial classification titles were selected. Characteristics of 268 industries, including, but not limited to, fabric finishing, wood preserving, pulp mills, industrial organic chemical manufacturing, and paint, rubber, and leather manufacturing, were compiled into a database. Industries were geocoded following the same matching procedure as described for residences: 89% matched to the address, 5% were placed on the road using best informed guess, and as a last resort, 6% were matched to town centroid. Each industry was assigned a start year and end year, based on best available data. The data on these industries is used to demonstrate the focused versions of the *Q *statistics.

## Results

At the time of this writing, geocoding and data collection are ongoing; hence the results reported in this manuscript are entirely preliminary and should not be used to draw any conclusions regarding the spatial patterns of bladder cancer in Michigan. The analysis undertaken in the manuscript is provided only as an example application of the new *Q *statistics.

To demonstrate the methods we implemented the local and global *Q *statistics for clustering of residential histories, specifically the local test at time *t*, *Q*_*i*,*k*,*t *_(Equations 6), and its global counterpart *Q*_*k*,*t *_(Equation 10). We also implemented the local test for clustering of residential histories through time *Q*_*i*,*k *_(Equation 13), and the global test for clustering of residential histories *Q*_*k *_(Equation 11). We also were concerned with possible clustering of cases near the industrial facilities, and evaluated this using the focused test at time *t Q*_*F*,*k*,*t *_(Equation 15) as well as the focused test through time *Q*_*F*,*k *_(Equation 16). In addition we programmed the duration-weighted versions of these statistics, and for the focused tests we also employed exposure weights calculated using the inverse rank distance (Equation 19).

### Results for Q_kt_

**Figure 3:**
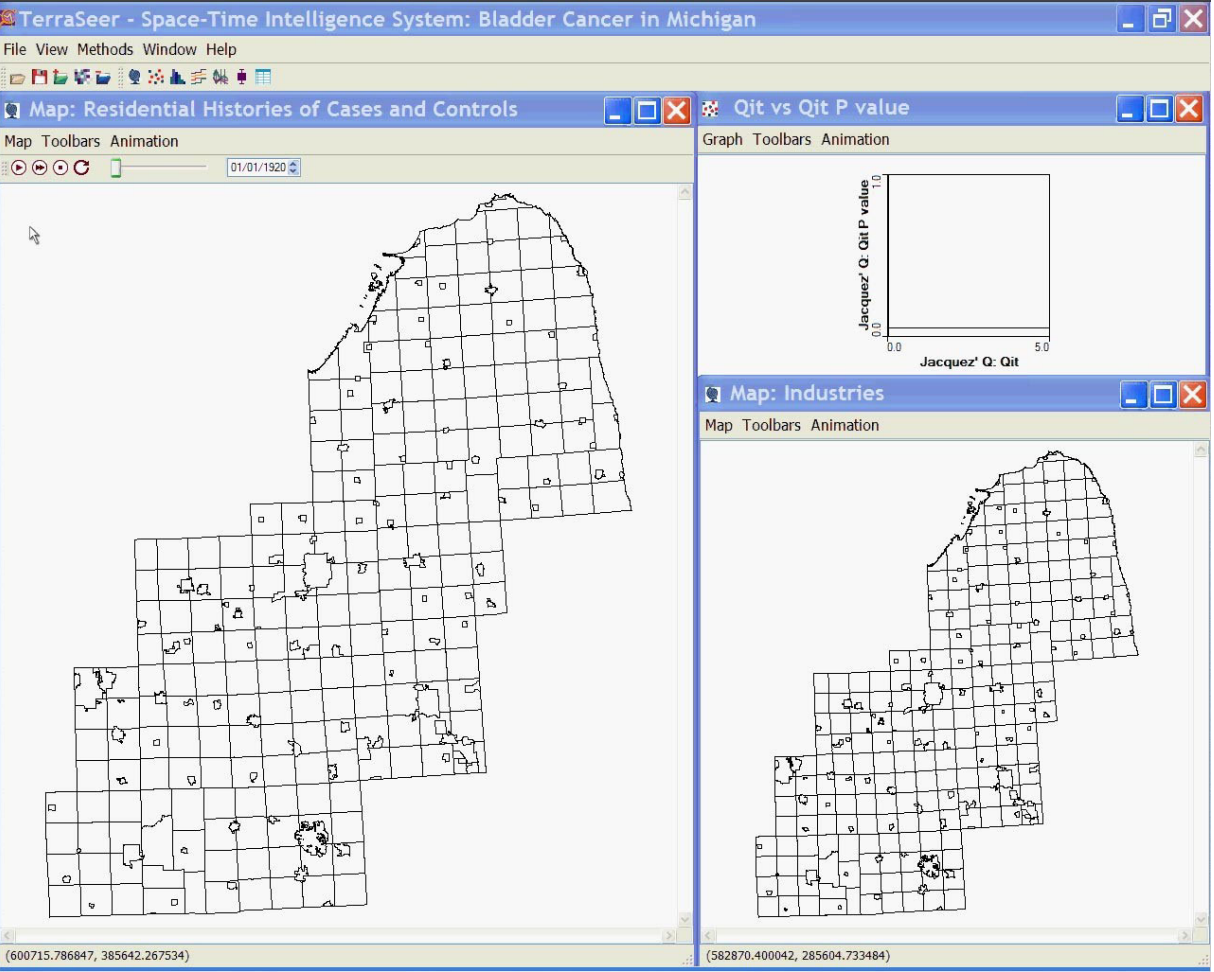
**Still from the animation of residential histories of cases, controls and industry in southeastern Michigan (**additional file 2**and**additional file 3**).**

These techniques were implemented in TerraSeer's STIS software using the Application Programmer's Interface. This allowed us to create a methods dynamic linked library with our new techniques that we then invoked using an automatically generated dialog. Time animated maps of the places of residence of the cases and controls, and of the changing geography of the municipal water supplies, were constructed using STIS (Figure 3 [see Additional file 2] [see Additional file 3]). These display the changing geography of the cases and controls as they move from one place to another, alterations in the geography of the municipal water supplies as they are founded, expand and merge, as well as township boundaries. To verify the methods we compared results using the *Q *statistics to those obtained using Cuzick and Edward's test in the ClusterSeer software. Specifically, we used STIS to calculate the *Q*_*kt *_statistics through time and then exported the data for July 1, 1969. We choose this time point, because *Q*_*kt *_reached a local peak of *Q*_*kt *_= 77 that was statistically significant (see Figure 1). The Cuzick and Edward's test in ClusterSeer returned T_5 _= 77, confirming the results from STIS. As noted earlier, Cuzick and Edward's test is a special case of the *Q*-statistic for the global test at time *t*, *Q*_*kt*_. Note that *Q*_*kt *_is calculated as the sum of the local *Q *statistics at time *t*, *Q*_*ikt*_, and thereby provides verification that the statistic *Q*_*ikt*_, from which the family of *Q *statistics is derived, is being calculated correctly. We must remind the reader that these results are highly preliminary and that data collection is incomplete. In fact, and as noted later in the Discussion, it is likely the observed clustering in these data is due to the geographic ordering in which the data are being collected. Nonetheless, this example demonstrates how plots of the *Q*_*kt *_statistics may be used to evaluate geographic case clustering of residential histories.

### Results for 

**Figure 4:**
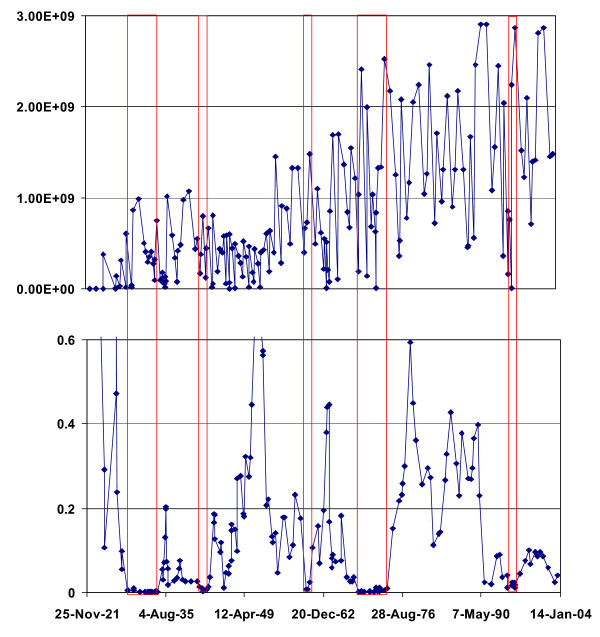
**through time**. Graph of  (top) and its probability (bottom) through time for *k *= 5.  is the time weighted version of Q_tki _and is expressed in case-seconds.

The results reported above were not time standardized. We therefore undertook an analysis using the time-standardized version of *Q*_*kt *_called  as per Equation A4. This expresses the amount of clustering at a given time interval in cases per unit time period. STIS reports times down to the second, hence results are recorded in person seconds. Figure 4 shows a similar overall increasing trend but also a greater variability in the value of the Q statistic through time. This is driven both by the increased number of cases through time and also by differences in the durations between movement events. When these sources of variability are accounted for we find episodic case clustering in approximately the same time intervals as found for the not time weighted statistic.

### Results for *Q*_*k*_

Having found some of the *Q*_*kt *_and  statistics to be statistically significant, the question then arises as to whether there is overall global clustering given the multiple time points evaluated. To accomplish this we used the global *Q*_*k *_test under the randomization procedure that holds the residential histories of the cases and controls as given and then allocates the *n*_*a *_case and *n*_*b *_control identifiers across the *N *residential histories. We accomplished this randomization 99 times in the STIS with a resulting p-value of 0.01, and concluded there was global clustering in the residential histories.

### Results for *Q*_*i*,*k *_to evaluate Clustering of Residential Histories

**Figure 5:**
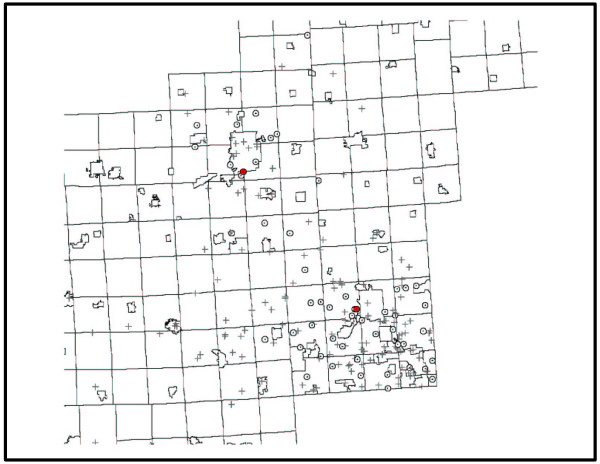
**Map of cases and controls on 4/12/1997**. Map of cases and controls on 4/12/1997. Cases are shown as dots within a circle, controls are shown as crosses. The two cases whose residential histories tend to be surrounded by the residential histories of other cases are shown in red.

The statistics *Q*_*kt *_and  are sensitive to a clustering of cases relative to the controls, and are evaluated at each of the *T*+1 time points in the set of residential histories. We also can ask, whether residential histories of the cases cluster near the residential histories of other cases by using the statistics *Q*_*i*,*k *_(Equation 13) and its duration-weighted version  (Equation A6). Since our analysis above demonstrated the results are not overly sensitive to duration weighting, we report results only for the not-weighted tests. This test will associate a statistic and a p-value with each residential history. A map of the residential histories on April 12, 1997 is shown in Figure 5. Note the two red dots that denote the place of residence of the two cases with statistically significant clustering of residential histories. Over the entire time span of the study, these two cases tend to be surrounded by residential histories of other cases, rather than the residential histories of controls. Because of residential mobility, the two red dots move about through time. This animation is quite compelling in the STIS and is approximated by the simpler animation in Figure 3. Note the animation in Figure 3 is sampled from the complete animation created when running the STIS software. This is necessary to create .avi files of small enough size for effective posting on the internet. Periods in which a red dot disappears from the animation denote time periods when that individual moved out of the study area. It is important to note that we have not adjusted these local tests for the multiple testing in the many spatial locations that were evaluated. However, the global *Q*_*k *_statistic was statistically significant and the large local statistics observed for the two red dots reference the two residential histories that contributed the most to the global *Q*_*k*_.

### Focused clustering

To demonstrate the use of the focused versions of the *Q *statistic we analyzed possible clustering of the residential histories of cases near the 268 industrial facilities that produced compounds thought to be putative carcinogens for bladder cancer. We undertook two sets of analyses using *Q*_*F*,*k *_(Equation 16). The first evaluated focused clustering of residential histories using *k *= 5 nearest neighbors. The second only considered those nearest neighbors within 1 kilometer of the focus.

When considering the 5 nearest neighbors to each industry, 24 of the 268 industrial facilities had p-values less than 0.05. Thus under the null hypothesis that each person in the study had an equal probability of being labeled a case, these 24 candidate foci had a significant excess of cases among each of their five nearest neighbors, at least at the nominal 0.05 level. Notice that at the 0.05 level, we would have expected 13.4 foci to be significant under this null hypothesis. Using an experiment-wise error approach, and a 5% critical value, the adjusted alpha level of the test is 0.000187 using the Bonferonni correction, and is 0.000191 using Sidak's multiplicative inequality. Using 49,999 randomizations, we were able to resolve p-values as small as 0.00005. None of these industries proved to be statistically significant foci once multiple testing was accounted for.

We also used the distance-based approach considering those neighbors within 4,000 m of each industrial facility. Under this approach, 10 industrial facilities had p-values < 0.05, but none of these were significant once multiple testing was accounted for.

## Discussion

This paper presented a new approach for evaluating case control clustering of residential histories. To date and to our knowledge, almost all case control cluster tests rely on the static view, analyzing clustering at one point in time or independently at several points in time. By using the mathematical construct of a residential history in Equation 1, and the notion of super sets of proximity matrices (Equation 5) to represent the changing geometry of place of residence, we have derived local, global and focused tests that are realistic in the sense that they quantify human residential mobility.

The results of the analysis of the bladder cancer data are entirely preliminary, and should not be interpreted to reach any inferences or conclusions regarding case-control clustering of bladder cancer in Michigan. At the time of this writing we believe statistically significant spatial clustering of cases is the result of a geographic pattern in the temporal order in which cases are reported. Because of recent implementation of the HIPPA (Health Insurance Portability and Accountability Act) legislation, The University of Michigan hospital systems had been unwilling to release case data until its official position on these requirements was completely formulated. As a result, bladder cancer cases that were treated at the University of Michigan hospitals are only now being recruited to the study's data set. Because many of these cases are from the surrounding environs of Washtenaw and Livingston counties, the data set analyzed in this paper has a deficit of cases in these areas. Selection of controls employs a population sample using random digit dialing, and appropriately represents the entire study area. As a result, there is a deficit of cases in Washtenaw and Livingston counties, and a concomitant clustering of cases in the balance of the study area. Further investigation of any bladder cancer clusters would also entail including known bladder cancer risk factors, such as cigarette smoking and occupational exposure history, in the analysis. We intend to revisit this analysis once the data set is complete.

Selection of controls through random digit dialing can introduce bias, since not everyone is equally likely to be selected due to different numbers of phones and the likelihood of answering the phone. While such bias might be reduced by first selecting a census block group based on census numbers, adjusted for age and gender, and then doing random digit dialing within that block group, such a procedure has the potential of over-matching on exposure [53]. This would make it very difficult to detect any spatial pattern that arises at a spatial scale greater than the block group. In this study we chose not to match on geography because some of the exposures of interest display a geographic pattern and over-matching on exposure was a possibility. These exposures include regional patterns in arsenic concentration in drinking waters associated with surficial geology and regional differences in household water supply sources [54,55].

Southeastern Michigan includes rural farming areas as well as portions of metropolitan Detroit, and differential response rates under random digit dialing are a concern. We attempted to ensure these areas do not have differential response rates by comparing addresses of responders and non-responders in age-weighted lists.

Exposures in early life and over an individual's life course may be important risk factors for the onset of cancer [56,57,34], thereby impacting both the date of diagnosis and latency period. But how can such risk factors be accounted for in exposure trace analysis? We need to explicitly model the latency period to take into account not only exposures of direct interest (arsenic in our example) but also additional risk factors (such as smoking) that might decrease the latency period and accelerate disease onset. Many common epidemiological risk-disease measures (e.g. odds ratio) are concerned with *whether *an exposure occurred, rather than with *when *it occurred, and are thus of little use for estimating relationships between timing of exposure and disease onset [58]. For cohort analyses, Robins and Greenland[59] argued that, when conditioned on age, Years of Life Lost (YLL) due to early exposures cannot be estimated without bias in the absence of causal models for how exposure causes death. This result was demonstrated analytically by Morfeld [60], who developed a framework for causal thinking in epidemiology, and applied it to evaluate the estimability of YLL and related measures. Candidate causal modeling approaches cited by Morfeld include Robin's G-estimation procedure [61,62] which can be used to estimate the time period between exposures and outcomes such as death, and thus appear promising for incorporating covariates into models of the latency period. Applications of G-estimation and the YLL procedures of Robins [61] in exposure trace modeling is thus an important future research direction.

Discussion of the type of spatial metric to use (nearest neighbor, adjacency, or geographic distance-based) as well as the number of *k *nearest neighbors to analyze is warranted. The approaches detailed in this paper are general in the sense that weights such as inverse distance and adjacency could be used in place of *k*-nearest neighbor relationships in Equation 4. We chose to work with nearest neighbor measures because we've found them to be more powerful than adjacency- and distance-based measures in some situations (e.g. [63]). As noted earlier in this paper, we used *k *= 5 because we found in the past that spatial clustering under nearest neighbor methods often may be detected at that level of *k*. Such a justification is sufficient in analyses conducted purely for demonstration purposes but is deficient in applied settings. In practice, two approaches may be used, which we call *a priori *and *exploratory*. When prior information is available on the scale of clustering this can be used to select a specific number of nearest neighbors to explore. Hence if one wishes to detect clusters of five individuals one might set *k *= 5. When such prior information is lacking an exploratory approach may be used in which several levels of *k *are analyzed, and probabilities from the analyses must then be adjusted to account for multiple testing [63].

We could not demonstrate each of the statistics developed in this paper, due to both data and space constraints. We note that exposure traces could be implemented to represent cases and controls of similar ages, in addition to those at a point in time. For example, a researcher may wish to determine whether cases cluster together when they were children, irrespective of year, thereby indicating early-lifetime vulnerability to an environmental exposure in the area. These clustering tools thus can be used to display cancer clusters of similarly-aged participants, as well as clusters based on the years a participant lived at a residence. In this manner, clusters of children can be investigated, whether they are born in the same generation or born in different generations.

## Conclusion

In conclusion, the methods presented in this paper account for residential mobility and are thus far more realistic than existing tests that are founded on static geographic representations. They thus are preferred over clustering methods that ignore human mobility. The techniques demonstrated in this paper have been programmed in a dynamic linked library that can be obtained from the first author and used in conjunction with a STIS.

## List of Abbreviations

GIS: Geographic Information System

HIPPA: Health Insurance Portability and Accountability Act

H_IV_: Goovaert and Jacquez's [45] neutral model Type IV

H_VI_: Goovaert and Jacquez's [45] neutral model Type IV

IRB: Institutional Review Board

LISA: Local Indicators of Spatial Autocorrelations

MCMC: Markov Chain Monte Carlo

STIS: Space-Time Intelligence System

SIC: Standard Industrial Classification code

YLL: Years of Life Lost

## Competing interests

Geoffrey Jacquez is President of BioMedware, the software company that is developing the STIS software.

## Authors' contributions

GJ derived the methods and drafted the majority of this manuscript. He also accomplished the analysis of the bladder cancer data set. AK programmed and tested the statistical methods in the STIS software. GA and JM provided data and wrote the data set description. PG wrote the sections on geostatistical weighting functions for the focused tests. JN is Principal Investigator on the R01 project that is collecting the bladder cancer data set.

## Supplementary Material

Additional File 1 **Appendix **This file contains the article's Appendix. Click here for file

Additional File 2 **Animation for Figure **3**in QuickTime **This is the animation for Figure 3 in QuickTime format. Click here for file

Additional File 3 **Animation for **Figure 3**as an animated GIF **This is the animation for Figure 3 in GIF format. Click here for file

## References

[B1] Collia DV, Sharp J, Giesbrecht L (2003). The 2001 National Household Travel Survey: a look into the travel patterns of older Americans. J Safety Res.

[B2] Klepeis NE, Nelson WC, Ott WR, Robinson JP, Tsang AM, Switzer P, Behar JV, Hern SC, Engelmann WH (2001). The National Human Activity Pattern Survey (NHAPS): a resource for assessing exposure to environmental pollutants. J Expo Anal Environ Epidemiol.

[B3] Reuscher TR, Schmoyer RL, Hu PS (2002). Transferability of Nationwide Personal Transportation Survey data to regional and local scales. Transport Res Rec.

[B4] Sabel CE, Boyle PJ, Löytönen M, Gatrell AC, Jokelainen M, Flowerdew R, Maasilta P (2003). Spatial clustering of amyotrophic lateral sclerosis in Finland at place of birth and place of death. Am J Epidemiol.

[B5] Syndromic surveillance in public health practice, New York City. http://www.cdc.gov/ncidod/EID/vol10no5/03-0646.htm.

[B6] Mather FJ, Whited LE, Langlois EC, Shorter CF, Swalm CM, Shaffer JG, Harley WR (2004). Statistical methods for linking health, exposure and hazards. Environ Health Perspect.

[B7] Pickle LW, Waller LA, Lawson AB Current practices in cancer spatial data analysis: a call for guidance. Int J Health Geogr.

[B8] Boscoe FP, Ward MH, Reynolds P (2004). Current practices in spatial analysis of cancer data: data characteristics and data sources for geographic studies of cancer. Int J Health Geogr.

[B9] Goodchild M (2000). GIS and transportation: status and challenges. GeoInformatica.

[B10] AvRuskin GA, Jacquez GM, Meliker JR, Slotnick MJ, Kaufmann A, Nriagu JO (2004). Visualization and exploratory analysis of epidemiologic data using using a novel space time information system. Int J Health Geogr.

[B11] Jacquez GM, Greiling D, Kaufmann A (2005). Design and implementation of a Space-Time Intelligence System for disease surveillance. J Geograph Syst.

[B12] Greiling DA, Jacquez GM, Kaufmann AM, Rommel RG (2005). Space time visualization and analysis in the Cancer Atlas Viewer. J Geograph Syst.

[B13] Meliker J, Slotnick M, AvRuskin GA, Kaufmann A, Jacquez GM, Nriagu JO (2005). Improving exposure assessment in environmental epidemiology: application of a spaho-temp oral visualization tools. J Geograph Syst.

[B14] Miller H (2005). A measurement theory for time geography. Geogr Anal.

[B15] Gustafson EJ (1998). Quantifying landscape spatial pattern: What is the state of the art?. Ecosystems.

[B16] Waller LA, Jacquez GM (1995). Disease models implicit in statistical tests of disease clustering. Epidemiology.

[B17] Lawson AB, Kulldorff M, Lawson A, Bohning D, Lesaffre E, Viel J-F, Bertollini R (1999). A review of cluster detection methods. Advanced Methods of Disease Mapping and Risk Assessment for Public Health Decision Making.

[B18] Jacquez GM, Grimson R, Waller L, Wartenberg D (1996). The analysis of disease clusters Part 2: Introduction to techniques. Infect Control Hosp Epidemiol.

[B19] Jacquez GM, Waller L, Grimson R, Wartenberg D (1996). The analysis of disease clusters Part I: State of the art. Infect Control Hosp Epidemiol.

[B20] Moran PA (1950). Notes on continuous stochastic phenomena. Biometrika.

[B21] Ord JK, Getis A (1995). Local spatial autocorrelation statistics: Distributional issues and an application. Geogr Anal.

[B22] Lawson AB (1989). Score tests for detection of spatial trend in morbidity data.

[B23] Waller LA, Turnbull BW, Clark LC, Nasca P (1992). Chronic disease surveillance and testing of clustering of disease and exposure: Application to leukemia incidence and TCE-contaminated dumpsites in upstate New York. Environmetrics.

[B24] Lawson AB, Waller LA (1996). A review of point pattern methods for spatial modelling of events around sources of pollution. Environmetrics.

[B25] Kulldorff M, Athas WF, Feuer E, Miller B, Key C (1998). Evaluation of cluster alarms: A space-time scan statistic and brain cancer in Los Alamos. Am J Public Health.

[B26] Zhan FB (2002). Are deaths from liver cancer, kidney cancer, and leukemia clustered in San Antonio?. Tex Med.

[B27] Roche LM, Skinner R, Weinstein RB (2002). Use of a geographic information system to identify and characterize areas with high proportions of distant stage breast cancer. J Public Health Manag Pract.

[B28] Gregorio DI, Kulldorff M, Barry L, Samociuk H (2002). Geographic differences in invasive and in situ breast cancer incidence according to precise geographic coordinates, Connecticut, 1991–95. Int J Cancer.

[B29] Jemal A, Kulldorff M, Devesa SS, Hayes RB, Fraumeni JF (2002). A geographic analysis of prostate cancer mortality in the United States. Int J Cancer.

[B30] Thomas A, Carlin BP (2003). Late detection of breast and colorectal cancer in Minnesota counties: An application of spatial smoothing and clustering. Stat Medicine.

[B31] Hsu CE, Jacobson HE, Mas FS (2004). Evaluating the disparity of female breast cancer mortality among racial groups; a spatiotemporal analysis. Int J Health Geogr.

[B32] Vieira V, Webster T, Weinberg J, Aschengrau A, Ozonoff D (2002). Spatial analysis of lung, breast and colorectal cancer on Cape Cod using generalized additive modeling [abstract]. Epidemiology.

[B33] Vieira V, Webster T, Aschengrau A, Ozonoff D (2002). A method for spatial analysis of risk in a population-based case-control study. Int J Hyg Environ Health.

[B34] Han D, Rogerson PA, Nie J, Bonner MR, Vena JE, Vito D, Muti P, Trevisan M, Edge SB, Freudenheim JL (2004). Geographic clustering of residence in early life and subsequent risk of breast cancer (United States). Cancer Causes Control.

[B35] Hagerstrand T (1970). What about people in regional science?. Pap Reg Sci.

[B36] Hornsby K, Egenhofer M (2002). Modeling moving objects over multiple granularities, special issue on Spatial and Temporal Granularity. Ann Math Artif Intell.

[B37] Miller HJ (1991). Modeling accessibility using space-time prism concepts within geographical information systems. IJGIS.

[B38] Huisman O, Forer P (1998). Computational agents and urban life spaces: A preliminary realization of the time-geography of student lifestyles. Proceedings of the 3rd International Conference on GeoComputation.

[B39] Moore AB, Whigham P, Holt A, Aldridge C, Hodge K A time geography approach to the visualisation of sport. Proceedings of the 7th International Conference on GeoComputation University of Southampton, United Kingdom, CD-ROM.

[B40] Kwan MP, Janelle DG, Hodge DC (2000). Extensibility and individual hybrid accessibility in space time: A multi scale representation using GIS. Information, Place and Cyberspace: Issues in Acessibility.

[B41] Kwan MP, Janelle DG, Goodchild MF (2003). Accessibility in space and time: A theme in spatially integrated social science. J Geograph Syst.

[B42] Sinha G, Mark DM (2005). Measuring similarity between geospatial lifelines in studies of environmental health. J Geograph Syst.

[B43] Cuzick J, Edwards R (1990). Spatial clustering for inhomogeneous populations. J R Stat Soc [Ser B].

[B44] Jacquez GM (1994). Cuzick and Edwards test when exact locations are unknown. Am J Epidemiol.

[B45] Goovaerts P, Jacquez G (2004). Accounting for regional background and population size in the detection of spatial clusters and outliers using geostatistical filtering and spatial neutral models: the case of lung cancer in Long Island, New York. Int J Health Geogr.

[B46] Liebisch N, Jacquez GM, Goovaerts P, Kaufmann A (2002). New methods to generate neutral images for spatial pattern recognition. Lect Notes Comput Sc.

[B47] Simes RJ (1986). An improved Bonferroni procedure for multiple tests of significance. Biometrika.

[B48] Small MJ, Nunn AR, Forslund BL, Daily DA (1995). Source attribution of elevated residential soil lead near a battery recycling site. Environ Sci Tech.

[B49] Goovaerts P (1997). Geostatistics for Natural Resources Evaluation.

[B50] Saito H, Goovaerts P (2001). Accounting for source location and transport direction into geostatistical prediction of contaminants. Environ Sci Tech.

[B51] Goovaerts P, Van Meirvenne M, Soares A, Gomez-Hernandez J, Froidevaux R (2001). Delineation of hazardous areas and additional sampling strategy in presence of a location-specific threshold. geoENV III – Geostatistics for Environmental Applications.

[B52] (2000). Toxics Release Inventory (TRI) Data Files. http://www.epa.gov/tri/tridata/tri00/data/index.htm.

[B53] Agudo A, Gonzalez CA (1999). Secondary matching: A method for selecting controls in case-control studies on environmental risk factors. Int J Epidemiol.

[B54] Goovaerts P, Avruskin G, Meliker J, Slotnick M, Jacquez G, Nriagu J Geostatistical Modeling of the Spatial Variability of Arsenic in Groundwater of Southeast Michigan. Water Resour Res Accepted.

[B55] Kolker A, Haack SK, Cannon WF, Westjohn DB, Kim MJ, Nriagu J, Woodruff LG, Welch AH, Stollenwerk KG (2003). Arsenic in southeastern Michigan. Arsenic in Ground Water.

[B56] Barker DJP (1992). Fetal and Infant Origins of Adult Disease.

[B57] Kuh D, Ben-Shlomo Y (1997). A Life Course Approach to Chronic Disease Epidemiology: Tracing the Origins of Ill-health from Early to Later Life.

[B58] Morfeld P (2003). Letter to the Editor. An alternate characterization of hazard in occupational epidemiology: Years of life lost per years worked. Am J Ind Med.

[B59] Robins JM, Greenland S (1991). Estimability and estimation of expected years of life lost due to a hazardous exposure. Stat Medicine.

[B60] Morfeld P (2004). Years of Life Lost due to Exposure – Causal Concepts and Empirical Shortcomings. Epidemiol Perspect Innov.

[B61] Robins JM, Berkane M (1997). Causal inference from complex longitudinal data. Latent variable modeling with applications to causality.

[B62] Rothmann KJ, Greenland S (1998). Modern Epidemiology.

[B63] Jacquez GM (1996). A k-nearest neighbor test for space-time interaction. Stat Medicine.

