# Supplementary material for: Global, local and focused geographic clustering for case-control data with residential histories
Source: Environ Health. 2005 Mar 22;4:4. doi: 10.1186/1476-069X-4-4 (PMC1083418; doi:10.1186/1476-069X-4-4)
Supplement: Additional File 1 — Appendix This file contains the article's Appendix. [file 1476-069X-4-4-S1.doc]

## Appendix

### Duration weighted tests

Define the duration of the *t*^th^ time period to be:

$\omega_{o}=(t_{o+1})-t_{o}$ (Equation A1)

Notice the change in notation from the body of this paper so that the times at which observations are made are now subscripted, allowing us to assign varying durations to the times between observations. A statistic, such as the local case control cluster statistic has a given value for the period from *t_o_* to *t_o+1_*. At time *t_o+1_* it takes on the new value defined by$Q_{i,k,t_{o+1}}$, and that value pertains until the next observation *o*+2. Notice the times when observations are made are separated by periods that are not necessarily of equal duration, so $\omega_{o}$ does not necessarily equal$\omega_{o+1}$. One then can define a duration weighted version of the local cluster statistic as

$Q_{i,k,\omega_{o}}=Q_{i,k,t_{o}} \omega_{o}$ (Equation A2)

The units on the duration weighted version are case-time units (e.g. case days). Each of the statistics defined previously can now be redefined as duration-weighted versions, with the proviso that all summations over time are from *o*=0 to *o*=*T*-1. Specifically, the duration weighted version of the local statistic is:

$Q_{i,k,\omega_{o}}=c_{i}\sum_{j=1}^{N} \eta_{i,j,k,t_{o}} c_{j} \omega_{o}$ (Equation A3)

The duration weighted global statistic is:

$Q_{k,\omega_{o}}=\sum_{i=1}^{N} Q_{i,k,\omega_{o}}$ (Equation A4)

The duration weighted global statistic for clustering of residential histories is:

$Q_{k}^{\omega}=\sum_{o=0}^{T-1} Q_{k,\omega_{o}}$ (Equation A5)

The duration weighted local statistic for clustering of residential histories is:

$Q_{i,k}^{\omega}=\sum_{o=0}^{T-1} Q_{i,k,\omega_{o}}$ (Equation A6)

The duration weighted focused statistic over period $\omega_{o}$is:

$Q_{F,k,\omega{}_{o}}=\sum_{j=1}^{N} \eta_{F,j,k,t_{o}} c_{j} \omega_{o}$ (Equation A7)

The duration weighted test for focused clustering of residential histories through time is:

$Q_{_{F,k}}^{\omega}=\sum_{o=0}^{T-1} Q_{F,k,\omega_{o}}$ (Equation A8)

The weighted focused test over duration $\omega_{t}$is:

$Q'_{F,k,\omega_{t}}=\sum_{j=1}^{N} w_{F,j,t_{o}} c_{j} \eta_{F,j,k,t_{o}} \omega_{o}$ (Equation A9)

The weighted focused test, duration-based, for residential histories through time is:

(Equation A10)

When observations are made at regular time points such that $\omega_{0}=\omega_{1}=...=\omega_{T-1}$ the not time weighted statistics may be used. When observations are recorded at irregular time intervals the duration-based statistics should be used. The not duration-weighted versions also can be used when one wishes to determine whether any of the *T*+1 configurations of cases and controls are spatially clustered.
