# Supplementary figures and images for: Global, local and focused geographic clustering for case-control data with residential histories
Source: Environ Health. 2005 Mar 22;4:4. doi: 10.1186/1476-069X-4-4 (PMC1083418; doi:10.1186/1476-069X-4-4)

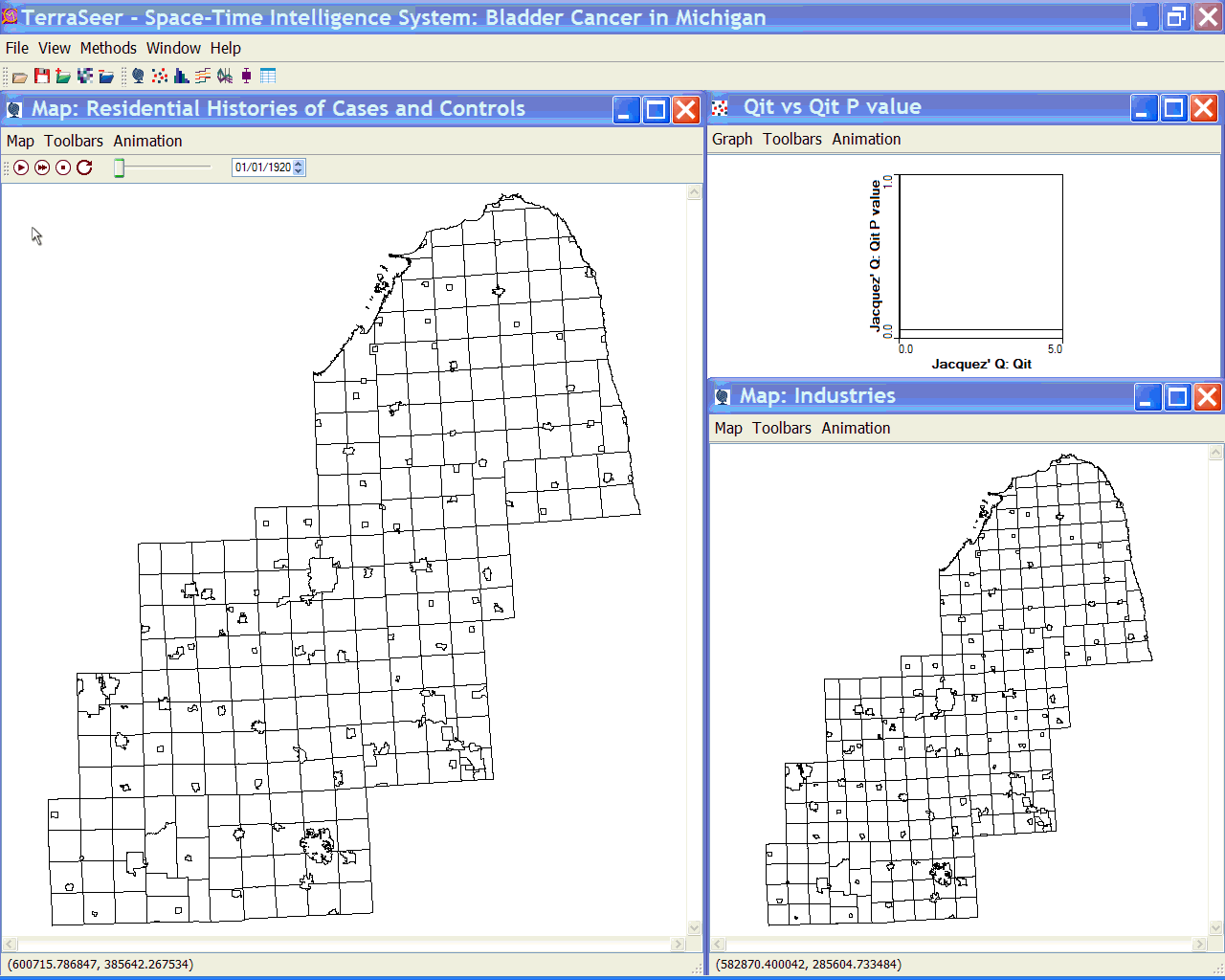

Supplement: Additional File 3 — Animation for Figure 3as an animated GIF This is the animation for Figure 3 in GIF format. [file 1476-069X-4-4-S3.gif]
